# Energy Insecurity and Mental Health Symptoms in US Adults

**DOI:** 10.1001/jamanetworkopen.2025.39479

**Published:** 2025-10-27

**Authors:** Michelle Graff, Ther W. Aung

**Affiliations:** 1 Carter School of Public Policy, Georgia Institute of Technology, Atlanta; 2 The MetroHealth System, School of Medicine, Case Western Reserve University, Cleveland, Ohio

## Abstract

**Importance:**

The national-scale mental health effects of energy insecurity—the inability to meet household energy needs—remain unknown. As energy insecurity becomes more prevalent in the US, these findings can help guide programs and health interventions aimed at addressing energy insecurity and reducing its health consequences.

**Objective:**

To investigate whether energy insecurity is associated with depression and anxiety symptoms among the US adult population.

**Design, Setting, and Participants:**

This study relied on repeated cross-sectional, nationally representative survey data from the US Census Bureau’s online Household Pulse Survey administered between December 9, 2022, and September 16, 2024. Eligible participants included US adults with email and mobile phone numbers.

**Exposures:**

Two exposure models were analyzed. One model included 3 energy insecurity components: (1) inability to pay full or partial energy bills, (2) keeping the home at unsafe or unhealthy temperatures; and (3) forgoing expenses for necessities, such as food and medicine, to pay energy bills. The second model included a composite measure for respondents who reported experiencing at least 1 of the 3 energy insecurity components in the past 12 months.

**Main Outcomes and Measures:**

Depression and anxiety symptoms were assessed using shortened versions of the Patient Health Questionnaire and Generalized Anxiety Disorder scale. The composite questionnaire scores were used to determine the respondents’ symptoms. Weighted bivariate and multivariable logistic regressions adjusting for sociodemographic characteristics were conducted.

**Results:**

The weighted sample included 187 356 336 US adults (45 551 454 [24.3%] aged 18-34 years; 96 305 955 [51.4%] female). A total of 81 176 522 adults (43.3%) reported experiencing at least 1 component of energy insecurity. Multivariable analyses showed that energy-insecure individuals had higher odds of reporting symptoms of depression and anxiety compared with those without energy insecurity. After adjusting for other social determinants of health, energy insecurity was associated with increased odds of anxiety (OR, 2.29; 95% CI, 2.24-2.34) and depression (OR, 2.31; 95% CI, 2.26-2.37) symptoms.

**Conclusions and Relevance:**

In this cross-sectional study of US adults, energy insecurity was associated with mental health symptoms. As household energy insecurity is expected to increase, these findings provide valuable evidence for policymakers deliberating the future of federally funded assistance programs.

## Key Points

**Question:**

Is household energy insecurity associated with symptoms of depression and anxiety among US adults?

**Findings:**

In this cross-sectional study of 187 million US adults, individuals experiencing at least 1 form of energy insecurity in the past year had significantly higher odds of reporting symptoms of depression and anxiety compared with individuals without energy insecurity.

**Meaning:**

These findings suggest that energy insecurity is a widespread and important factor associated with mental health symptoms and may warrant consideration in efforts to reduce adverse mental health outcomes.

## Teaser

This cross-sectional study examines whether indicators of energy insecurity are associated with symptoms of anxiety and depression among the US adult population.

## Introduction

Residential energy consumption in the US is rising, driven in part by increasing temperatures due to climate change.^1,2^ These changes may disproportionately affect low-income populations, as the need to maintain indoor comfort under more extreme conditions can raise energy costs.^3^ As a result, energy insecurity, or the inability of households to meet basic energy needs (eg, heating, cooling, cooking, and refrigeration), has emerged as an important public health concern.^4^ Energy insecurity is often measured through a combination of indicators, including financial challenges, such as difficulty paying energy bills^5^ or needing to sacrifice essentials like food and medicine,^6^ and behavioral responses, such as keeping homes at unsafe or uncomfortable temperatures to reduce costs.^7,8^ Research documents disparities in US energy insecurity, with households of racial minority groups, families with young children, and individuals reliant on electronic medical devices facing the highest rates of the material hardship.^9,10^

Despite its prevalence—affecting 27% of the US population in 2020^11^—energy insecurity and its consequences are understudied, especially at the national level. Researchers argue the gap in knowledge exists because the federal government does not classify energy insecurity distinctly from general poverty,^12^ which has limited its inclusion in national survey instruments. Therefore, much of the current research linking energy insecurity to health outcomes has been localized or regional. National studies connecting energy insecurity to health outcomes have relied on neighborhood-level income measures, rather than individual- or household-level estimates.^13,14^

Still, a growing body of evidence demonstrates energy insecurity is associated with poor health outcomes.^15^ For example, local studies^16,17^ link household energy insecurity with increased hospitalizations and poorer health in children. A survey in northern Manhattan^18^ indicated that households with energy insecurity were more likely to experience poor respiratory and sleep outcomes in adults. In 2024, Siegel et al^19^ found the 30% of New York City residents who experienced energy insecurity also had higher odds of being dependent on electronic medical devices and reported higher rates of poor respiratory and cardiovascular conditions.

However, the association between household energy insecurity and mental health remains poorly understood on a national scale. We contend that energy insecurity may contribute to chronic stress through persistent financial strain and the inability to meet basic household needs.^20^ Families who struggle to pay energy bills or are forced to endure unsafe indoor temperatures may face persistent physical discomfort and mental instability.^21^ These challenges are often compounded by trade-offs, such as forgoing food, medication, or medical care, which could further strain emotional well-being.^22^ Therefore, we expect these stressors will elevate the risk of anxiety and depression.

To address this gap, we relied on nationally representative data from the US Census Bureau’s Household Pulse Survey (HPS), initiated during the COVID-19 pandemic to track household well-being.^23^ Notably, the HPS asks questions on energy insecurity as well as health measures, allowing for a national analysis of the association of energy insecurity with mental health outcomes. Thus, we examined whether indicators of energy insecurity may be associated with symptoms of anxiety and depression among US adults. The results of the study can inform residential energy assistance policies^24^ and health care practices to improve the health of US residents who are likely to face higher energy insecurity risks due to a combination of increasing costs of living and rising outdoor temperatures.^25^

## Methods

The HPS sample is drawn from approximately 130 million housing units derived from the US Census Bureau’s Master Address File with matching phone and email addresses.^26,27^ Adults in selected households are invited via text and email to complete a survey online, with 1 individual per residence providing household responses to the questionnaire. When combined with survey weights provided by the HPS, the results provide nationally representative estimates of US households. The data used in this study are deidentified and publicly available; therefore, the study was exempt from institutional review board approval and the need for informed consent, in accordance with 45 CFR §46. The study followed the Strengthening the Reporting of Observational Studies in Epidemiology (STROBE) reporting guideline for cross-sectional studies.

### Outcomes

Depression and anxiety outcomes were assessed from a modified version of the 2-item Patient Health Questionnaire (PHQ-2) and the 2-item Generalized Anxiety Disorder scale (GAD-2), respectively.^28^ Both questionnaires ask about symptoms in the past 2 weeks. The PHQ-2 asks: “Over the last 2 weeks, how often have you been bothered by: (1) having little interest or pleasure in doing things; and (2) feeling down, depressed, or hopeless?” The GAD-2 asks: “How often have you been bothered by: (1) feeling nervous, anxious, or on edge; and (2) not being able to stop or control worrying?” Response options included not at all (coded 0), several days (coded 1), more than half the days (coded 2), and nearly every day (coded 3). Responses were summed for each scale, with a total score of 3 or greater on the PHQ-2 or GAD-2 considered indicative of a positive screen for major depressive disorder and generalized anxiety disorder symptoms, respectively.^29,30^

### Exposures

Exposures of interest were 3 binary indicators of energy insecurity experienced by households in the past 12 months. Indicators were constructed from responses to the following questions: (1) “How many times was your household unable to pay an energy bill or unable to pay the full bill amount?” (2) “How many months did your household keep your home at a temperature that you felt was unsafe or unhealthy?” and (3) “How many months did your household reduce or forgo expenses for basic household necessities, such as medicine or food, in order to pay an energy bill?” For each question, we constructed a binary indicator in which respondents who selected “almost every month,” “some months,” or “1 or 2 months” were coded as having energy insecurity (1), while those who answered “never” were coded as having energy security (0). Finally, we constructed a composite energy insecurity indicator if a respondent was classified with energy insecurity on any of the above component indicators.

### Covariates

We controlled for other social determinants of health (SDOHs) to help contextualize the role of energy insecurity. Given that households experiencing energy insecurity often face compounding SDOHs,^31^ we included measures of unemployment, housing instability, and food insecurity.

Other covariates in the analyses were age, educational level, sex, annual household income, self-reported race and ethnicity, marital status, household size, number of children in the home, home type, home ownership, functional disability, census region, survey week, and presence of time-based utility disconnection policy in each state during each time period. Race and ethnicity were assessed because Black and Hispanic populations have higher rates of energy insecurity compared with White populations^5^; categories included Asian, Black, Hispanic, White, and other race or ethnicity (which included American Indian or Alaska Native, Chamorro, Native Hawaiian or Other Pacific Islander, Samoan, and multiracial individuals).

Last, we included data on utility disconnection policies from the Utilities Disconnection Dashboard.^32^ We coded each state and time period as having policy protection from utility disconnection if a date-based utility shutoff moratorium was in effect—that is, if state law prohibited regulated utilities from disconnecting residential power during a specific time frame. Currently, 24 states have date-based disconnection protections, with Connecticut implementing the most expansive policy: utilities cannot terminate service between November 1 and May 1.

All variables, except survey week and policy protection from utility disconnection, were self-reported. More information on covariate coding and definitions is provided in eMethods in Supplement 1.

### Statistical Analysis

The HPS data were pooled across 21 independent survey samples between December 9, 2022, and September 16, 2024. The mean weighted response rate across the 21 data collection periods was 6.4%, and the mean (SD) number of respondents was 68 873 (5440) per period.^26,27^ All estimates used person-weights provided by the US Census Bureau to generate nationally representative estimates. For variance estimation, we applied 80 sets of replicate weights—also provided by the US Census Bureau—using a balanced repeated replication method with the Fay adjustment value^33^ set to 0.5 to account for geographic clustering. For bivariable analyses, we used the Rao-Scott correction for the weighted χ^2^ test when assessing the association between mental health symptoms and each exposure variable and covariates (unemployed during the past week, housing instability, food insecurity during the past week, age, educational level, sex, annual household income, race and ethnicity, marital status, household size, children in the home, housing type, home ownership, disability, census region, survey week, and policy protection from utility disconnection).

We estimated 2 sets of multivariable logistic regression models for each mental health outcome. The first set of models included all individual indicators of energy insecurity: inability to pay energy bills, keeping the home at an unsafe temperature, and forgoing expenses to pay energy bills. The second set of models included a composite energy insecurity indicator representing individuals who experienced any form of energy insecurity. Both sets of models controlled for additional SDOHs and covariates.

We conducted 2-sided tests and considered *P* < .05 as statistically significant. For our analysis, we used on Stata, version 18 (StataCorp LLC).

## Results

The study sample included 1 139 607 respondents representing a weighted population size of 187 356 336 US adults (45 551 454 [24.3%] aged 18-34 years; 96 305 955 [51.4%] female, 91 050 381 [48.6%] male). Weighted measures are shown in Table 1. In terms of race and ethnicity, 9 456 479 respondents (5.0%) were Asian, 19 085 944 (10.2%) were Black, 30 125 654 (16.1%) were Hispanic, 120 709 420 (64.4%) were White, and 7 978 838 (4.3%) were of other race or ethnicity. Less than one-quarter of respondents (43 294 903 [23.1%]) had an annual income of less than $35 000.

**Table 1.** Weighted Sociodemographic Characteristics of the Study Population

Characteristic	Weighted No. (%) (N = 187 356 336)^a^
Outcome variables	
Depression symptoms^b^	34 082 583 (18.2)
Anxiety symptoms^c^	43 779 826 (23.4)
Energy-related SDOH	
Inability to pay energy bill	42 029 443 (22.4)
Keeping home at unsafe temperature	41 126 954 (22.0)
Forgoing expenses to pay energy bill	63 009 461 (33.6)
Composite energy insecurity indicator^d^	81 176 522 (43.3)
Other SDOH	
Unemployed during past week	69 587 511 (37.1)
Housing instability	14 242 333 (7.6)
Food insecurity during past week	20 327 361 (10.8)
Age, y	
18-34	45 551 454 (24.3)
35-49	50 446 614 (26.9)
50-64	47 979 322 (25.6)
≥65	43 378 946 (23.2)
Sex	
Female	96 305 955 (51.4)
Male	91 050 381 (48.6)
Race and ethnicity	
Asian	9 456 479 (5.0)
Black	19 085 944 (10.2)
Hispanic	30 125 654 (16.1)
White	120 709 420 (64.4)
Other^e^	7 978 838 (4.3)
Educational level	
High school or less	63 801 905 (34.1)
Some college or associate’s degree	55 244 376 (29.5)
Bachelor’s or graduate degree	68 310 055 (36.5)
Annual household income, $	
<35 000	43 294 903 (23.1)
35 000-74 999	54 684 819 (29.2)
75 000-149 999	55 414 463 (29.6)
>150 000	33 962 151 (18.1)
Marital status	
Married	106 697 729 (56.9)
Widowed, divorced, or separated	34 814 108 (18.6)
Never married	45 844 499 (24.5)
Household size, No. of persons	
1-2	82 117 738 (43.8)
3-4	70 453 707 (37.6)
≥5	34 784 891 (18.6)
No. of children in home	
None	120 991 383 (64.6)
1-2	52 136 096 (27.8)
≥3	14 228 858 (7.6)
Home type	
Detached 1-family	127 109 668 (67.8)
Attached 1-family	13 935 829 (7.4)
Apartment	35 468 175 (18.9)
Other^f^	10 842 664 (5.8)
Home ownership	
Owner	131 071 314 (70.0)
Renter	56 285 022 (30.0)
No. of severe disabilities	
None	160 967 724 (85.9)
1	19 788 916 (10.6)
≥2	6 599 696 (3.5)
Region	
Northeast	31 568 870 (16.8)
South	71 642 032 (38.2)
Midwest	39 210 375 (20.9)
West	44 935 058 (24.0)
Policy protection from utility disconnection	
No	158 665 456 (84.7)
Yes	28 690 880 (15.3)

Abbreviation: SDOH, social determinants of health.

^a^ Due to poststratification estimation, subgroup totals may not match total population.

^b^ Depression symptoms during the last 2 weeks were assessed via the 2-item Patient Health Questionnaire.

^c^ Anxiety symptoms during the last 2 weeks were assessed via the 2-item Generalized Anxiety Disorder scale.

^d^ Defined as having any of the 3 energy insecurity components (inability to pay energy bill, keeping home at unsafe temperature, or forgoing expenses to pay energy bills).

^e^ Includes American Indian or Alaska Native, Chamorro, Native Hawaiian or Other Pacific Islander, Samoan, and multiracial.

^f^ Includes mobile home, boat, and recreational vehicle.

One-fifth of the population reported they were unable to pay an energy bill (42 029 443 [22.4%]). Similarly, 41 126 954 respondents (22.0%) kept home temperatures at unsafe or unhealthy levels due to cost concerns. A higher prevalence of households—63 009 461 (33.6%)—reported forgoing expenses, such as food or medicine, to pay an energy bill. The composite energy insecurity indicator revealed 81 176 522 US adults (43.3%) reported experiencing at least 1 form of energy insecurity in the past year. In comparison, 69 587 511 adults (37.1%) reported being unemployed during the past week and 20 327 361 (10.8%) reported being food insecure in the past week, and 14 242 333 (7.6%) noted housing instability.

Bivariate unadjusted analyses (Table 2) illustrate that experiencing any energy insecurity indicator, including the composite measure, was associated with depression and anxiety symptoms. Similarly, experiencing other SDOHs, including housing instability and food insecurity, was associated with mental health symptoms.

**Table 2.** Weighted Bivariate Analyses of Depression and Anxiety Symptoms Among Survey Respondents

Characteristic	Weighted No. (%) (N = 187 356 336)^a^	
	Depression symptoms (n = 34 082 583)^b^	Anxiety symptoms (n 43 779 826)^c^
Energy-related SDOH		
Inability to pay energy bill		
No	19 884 704 (13.7)	26 675 978 (18.4)
Yes	14 197 879 (33.8)	17 103 848 (40.7)
Keeping home at unsafe temperature		
No	20 250 742 (13.8)	27 528 406 (18.8)
Yes	13 831 841 (33.6)	16 251 420 (39.5)
Forgoing expenses to pay energy bill		
No	13 963 283 (11.2)	19 395 723 (15.6)
Yes	20 119 300 (31.9)	24 384 103 (38.7)
Composite energy insecurity indicator^d^		
No	10 291 066 (9.7)	14 745 033 (13.9)
Yes	23 791 517 (29.3)	29 034 793 (35.8)
Other SDOH		
Unemployed during past week		
No	20 401 146 (17.3)	27 463 038 (23.3)
Yes	13 681 437 (19.7)	16 316 787 (23.4)
Housing instability		
No	29 030 267 (16.8)	37 783 884 (21.8)
Yes	5 052 316 (35.5)	5 995 942 (42.1)
Food insecurity during past week		
No	24 886 395 (14.9)	33 260 761 (19.9)
Yes	9 196 188 (45.2)	10 519 065 (51.7)
Age, y		
18-34	12 521 699 (27.5)	15 797 261 (34.7)
35-49	9 560 907 (19.0)	12 964 326 (25.7)
50-64	7 683 692 (16.0)	9 831 101 (20.5)
≥65	4 316 285 (10.0)	5 187 139 (12.0)
Sex		
Female	18 249 579 (18.9)	25 612 170 (26.6)
Male	15 833 004 (17.4)	18 167 656 (20.0)
Race and ethnicity		
Asian	1 287 974 (13.6)	1 447 865 (15.3)
Black	3 580 252 (18.8)	4 225 113 (22.1)
Hispanic	6 206 714 (20.6)	7 564 287 (25.1)
White	21 035 294 (17.4)	28 082 162 (23.3)
Other^e^	1 972 349 (24.7)	2 460 399 (30.8)
Educational attainment		
High school or less	14 106 555 (22.1)	16 257 623 (25.5)
Some college or associate’s degree	11 939 225 (21.6)	15 143 017 (27.4)
Bachelor’s or graduate degree	8 036 803 (11.8)	12 379 186 (18.1)
Annual household income, $		
<35 000	12 322 972 (28.5)	14 264 846 (32.9)
35 000-74 999	11 157 905 (20.4)	13 982 429 (25.6)
75 000-149 999	7 906 097 (14.3)	11 050 863 (19.9)
≥150 000	2 695 609 (7.9)	4 481 689 (13.2)
Marital status		
Married	14 102 137 (13.2)	19 735 395 (18.5)
Widowed, divorced, or separated	7 271 531 (20.9)	8 776 468 (25.2)
Never married	12 708 914 (27.7)	15 267 963 (33.3)
Household size, No. of persons		
1-2	13 647 767 (16.6)	17 269 182 (21.0)
3-4	13 055 357 (18.5)	17 127 541 (24.3)
≥5	7 379 459 (21.2)	9 383 103 (27.0)
No. of children in home		
None	21 840 433 (18.1)	27 135 277 (22.4)
1-2	9 389 323 (18.0)	12 780 102 (24.5)
≥3	2 852 826 (20.0)	3 864 446 (27.2)
Home type		
Detached 1-family	20 179 192 (15.9)	26 517 128 (20.9)
Attached 1-family	2 504 368 (18.0)	3 247 570 (23.3)
Apartment	8 576 761 (24.2)	10 685 730 (30.1)
Other^f^	2 822 263 (26.0)	3 329 398 (30.7)
Home ownership		
Owner	19 301 726 (14.7)	25 545 623 (19.5)
Renter	14 780 857 (26.3)	18 234 203 (32.4)
No. of severe disabilities		
None	22 370 145 (13.9)	30 555 338 (19.0)
1	7 647 983 (38.6)	8 896 157 (45.0)
≥2	4 064 455 (61.6)	4 328 331 (65.6)
Census region		
Northeast	5 232 362 (16.6)	6 970 519 (22.1)
South	13 791 670 (19.3)	17 379 848 (24.3)
Midwest	6 844 156 (17.5)	8 880 541 (22.6)
West	8 214 394 (18.3)	10 548 918 (23.5)
Policy protection from utility disconnection		
No	28 998 566 (18.3)	37 152 908 (23.4)
Yes	5 084 017 (17.7)	6 626 918 (23.1)

Abbreviation: SDOH, social determinants of health.

^a^ Each cell contains a row percentage, and denominators vary by variable. *P* < .01 for all comparisons except for unemployed past week (*P* = .43) and policy protection from utility disconnection (*P* = .17) with anxiety symptoms.

^b^ Depression symptoms during the last 2 weeks were assessed via the 2-item Patient Health Questionnaire.

^c^ Anxiety symptoms during the last 2 weeks were assessed via the 2-item Generalized Anxiety Disorder scale.

^d^ Defined as having any of the 3 energy insecurity components (inability to pay energy bill, keeping home at unsafe temperature, or forgoing expenses to pay energy bills).

^e^ Includes American Indian or Alaska Native, Chamorro, Native Hawaiian or Other Pacific Islander, Samoan, or multiracial.

^f^ Includes mobile home, boat, and recreational vehicle.

**Figure 1.**
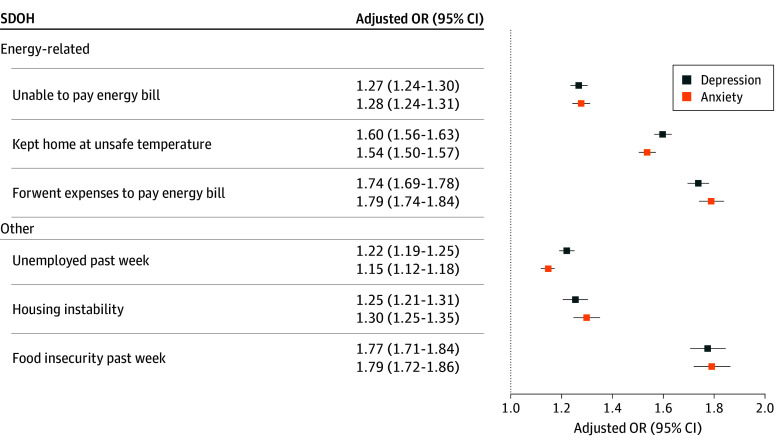
Multivariable Analysis of Mental Health, Energy Insecurity Components, and Social Determinants of Health (SDOH) Adjusted odds ratios (ORs) are estimated from weighted multivariable logistic regression models (weighted number of observations, 187 356 336). The models also include all respondent sociodemographic characteristics (age, educational level, sex, annual household income, race and ethnicity, marital status, household size, children in the home, home type, home ownership, disability, region, survey week, and policy protection from utility disconnection). Full model estimates are provided in eTable 1 in Supplement 1.

Our first multivariable models estimated the association between each energy insecurity component and mental health symptoms (Figure 1 and eTable 1 in Supplement 1). In these adjusted models, being unable to pay an energy bill, keeping indoor home temperatures at unsafe levels, and forgoing necessities to pay an energy bill were associated with increased odds of anxiety and depression symptoms compared with not experiencing any of these conditions. For example, those who forwent expenses to pay energy bills had higher odds of reporting anxiety (odds ratio [OR], 1.79; 95% CI, 1.74-1.84) and depression (OR, 1.74; 95% CI, 1.69-1.78) symptoms compared with those who did not forgo expenses for energy bill payments.

**Figure 2.**
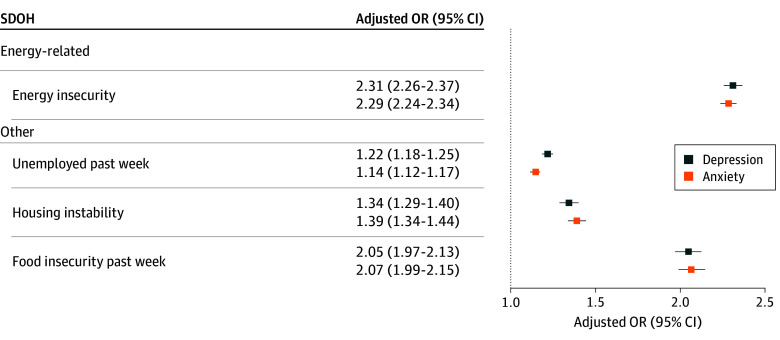
Multivariable Analysis of Mental Health, Composite Energy Insecurity Indicator, and Social Determinants of Health (SDOH) Adjusted odds ratios (ORs) are estimated from weighted multivariable logistic regression models (weighted number of observations, 187 356 336). The models also include all respondent level sociodemographic characteristics (age, educational level, sex, annual household income, race and ethnicity, marital status, household size, children in the home, home type, home ownership, disability, region, survey week, and policy protection from utility disconnection). Full model estimates are provided in eTable 2 in Supplement 1. The composite energy insecurity indicator is defined as having any of the 3 energy insecurity components (inability to pay energy bill; keeping the home at unsafe temperature; and forgoing expenses to pay energy bills).

The second set of adjusted multivariable models, which include the composite energy insecurity indicator, found respondents who experienced at least 1 energy insecurity condition in the past year had higher odds of experiencing depression (OR, 2.31; 95% CI, 2.26-2.37) and anxiety (OR, 2.29; 95% CI, 2.24-2.34) symptoms compared with those who did not report any energy insecurity conditions (Figure 2 and eTable 2 in Supplement 1). These models also reveal other SDOHs, including unemployment, housing instability, and food insecurity, were associated with mental health symptoms compared with being employed, having stable housing, or having food security. Among these, food insecurity was associated with higher odds of depression (OR, 2.05; 95% CI, 1.97-2.13) and anxiety (OR, 2.07; 95% CI, 1.99-2.15) symptoms compared with no food insecurity.

The sociodemographic covariates in the multivariable models associated with depression and anxiety symptoms were largely consistent across models (eTables 1 and 2 in Supplement 1). Adjusted estimates reveal unmarried respondents, those from larger households, tenants, and those with disabilities had higher odds of anxiety and depression symptoms compared with married respondents, those from the smallest households (1-2 persons), homeowners, and those without any disability. Living in a state with active date-based utility protection was not associated with symptoms of anxiety or depression compared with living in states without such a policy.

## Discussion

This nationally representative cross-sectional study found that energy insecurity was associated with mental health symptoms among US adults. One-third of respondents reported sacrificing necessities to afford energy costs, and approximately 22% indicated they were unable to pay an energy bill or had to endure unsafe indoor temperatures. More than 40% of respondents reported experiencing at least 1 form of energy insecurity in the past year. For context, in 2020—the most recent comparable data—the Residential Energy Consumption Survey conducted by the Energy Information Administration found 20% of US households had to forgo necessities to pay energy bills, 10% kept their homes at unsafe temperatures, and 27% reported experiencing general energy insecurity.^11^ Our more recent data offer a descriptive comparison, suggesting that energy insecurity in the US may have increased over time. Furthermore, respondents who reported experiencing at least 1 indicator of energy insecurity in the past year had higher odds of exhibiting symptoms of depression and anxiety compared with those experiencing energy security.

While our models do not allow for direct statistical comparisons across components of energy insecurity, the findings suggest each component may have different implications for mental health. For instance, maintaining a home at unsafe temperatures may create immediate physical discomfort and stress, whereas being unable to pay an energy bill or forgoing other necessities reflects financial strain and difficult tradeoffs. These distinctions may require targeted or layered solutions. For instance, in the short run, financial assistance can help households afford energy costs,^34^ while in the long run, investments in weatherization can ensure more comfortable indoor temperatures and reduce cost pressures.^35,36^

Extant scholarship documents the health effects of food insecurity^37,38,39^ and housing instability,^40,41,42,43^ while evidence on health effects of energy insecurity is relatively limited. This study’s descriptive findings indicate the prevalence of energy insecurity was higher (43.3%) than the prevalence of food insecurity (10.8%), suggesting energy insecurity may be an important factor to consider in SDOH-related mental health outcomes. Further, energy insecurity may act as a precursor to food insecurity, as our study found one-third of US adults forgo basic needs, including food, to pay energy bills. We recommend future studies investigate direct and indirect mechanisms linking energy insecurity to health outcomes to inform interventions and improve health in vulnerable populations.

Designing mechanistic studies and appropriate interventions will require more systematic and comprehensive individual-level data collection efforts. An increasing number of health systems conduct SDOH screening; however, only half of them screen for energy insecurity indicators.^44^ Wider implementation and harmonization of SDOH screening that captures energy insecurity with other SDOHs could generate the data necessary for health care professionals to identify and target households most at risk, thereby facilitating direct referrals to energy assistance programs and informing emerging clinical interventions. For example, “food as medicine” programs^45^ recognize the role of food access in promoting health among vulnerable populations.^46,47^ Expanding such interventions to help patients experiencing food and energy insecurity simultaneously may offer more sustainable improvements in health than practices targeting food insecurity alone.

Additionally, there is a need for more systematic and frequent population-level data collection on energy insecurity in national surveys, as existing federal instruments remain limited. For example, the American Community Survey collects household energy consumption data but does not assess households’ ability to pay energy bills, ability to maintain comfortable temperatures, or need to forgo expenses to pay for energy costs.^48^ The American Housing Survey includes a question on energy bill delinquency, but it is collected once every 4 years.^49^ The most comprehensive federal source on the topic is the Residential Energy Consumption Survey, and it is only collected once every 4 years and does not collect data on health or other key societal outcomes.^11^ As a result, there remains little national-level evidence linking household energy insecurity to physical or mental health, constraining the ability to inform policy and health care interventions.

### Strengths and Limitations

Our analysis benefited from its nationally representative HPS sample, which enhances generalizability, and high-frequency data collection, which likely reduces recall bias by asking respondents to report on more recent experiences, thereby improving accuracy.^50^ Still, the study has limitations. First, the HPS has a low response rate, although the US Census Bureau applies weighting adjustments to minimize nonresponse bias.^50^ It is possible that individuals experiencing energy insecurity or mental health symptoms may be less likely to participate in the HPS. For example, those unable to pay an electricity bill may also have limited or no access to the internet. If this is the case, our findings of prevalence on energy insecurity and associations with mental health are likely to be underestimated. Second, the observational and cross-sectional nature of the data limits causal inference and raises the possibility of reverse causality. Third, the questionnaire captured subjective interpretations of unsafe or unhealthy indoor temperature, which may vary among respondents. Fourth, variable recall periods differ: energy insecurity was assessed during the past 12 months, whereas mental health outcomes captured symptoms in the past 2 weeks. Despite these limitations, the HPS provides an opportunity to examine household-level energy insecurity and mental health on the national scale.

## Conclusions

This cross-sectional study offers nationally representative evidence that difficulty affording household energy needs may be associated with increased symptoms of anxiety and depression among US adults. Despite its high prevalence, energy insecurity remains underrecognized in public health and policy intervention strategies. Expanding, coordinating, and better publicizing efforts that enhance household energy affordability, such as bill assistance, payment plans, and weatherization services,^51^ may offer a meaningful strategy to support mental health and promote household stability amid rising energy costs and climate-related stressors. Last, because residential energy assistance is often underfunded, programs typically prioritize households whose members have physical health risks, such as young children, older adults, and individuals with disabilities.^52^ Our findings suggest that these priorities could be broadened to include households facing mental health challenges to better reach those most at risk.

## Notes

Supplement 1. **eMethods.** Definitions and Measurements **eTable 1.** Multivariable Analyses of Association Between Energy Insecurity Components and Mental Health Symptoms **eTable 2.** Multivariable Analyses of Association Between Composite Energy Insecurity Indicator and Mental Health Symptoms

Supplement 2.
Data Sharing Statement
