# Supplementary material for: Energy Insecurity and Mental Health Symptoms in US Adults
Source: JAMA Netw Open. 2025 Oct 27;8(10):e2539479. doi: 10.1001/jamanetworkopen.2025.39479 (PMC12559964; doi:10.1001/jamanetworkopen.2025.39479)
Supplement: Supplement 1. — eMethods. Definitions and Measurements eTable 1. Multivariable Analyses of Association Between Energy Insecurity Components and Mental Health Symptoms eTable 2. Multivariable Analyses of Association Between Composite Energy Insecurity Indicator and Mental Health Symptoms [file jamanetwopen-e2539479-s001.pdf]

Supplementary Online Content

Graff M, Aung TW. Energy insecurity and mental health symptoms in US adults. *JAMA Netw Open.* 2025;8(10):e2539479. doi:10.1001/jamanetworkopen.2025.39479

eMethods. Definitions and Measurements
eTable 1. Multivariable Analyses of Association Between Energy Insecurity Components and Mental Health Symptoms
eTable 2. Multivariable Analyses of Association Between Composite Energy Insecurity Indicator and Mental Health Symptoms

This supplementary material has been provided by the authors to give readers additional information about their work.

© 2025 Graff M and Aung TW. *JAMA Network Open*.

eMethods. Definitions and Measurements

We provide additional information on covariate definitions here. First, we provide details on how the Household Pulse Survey (HPS) asks respondents about social determinants of health (SDOH). Unemployment was captured from a binary question, “In the last 7 days, did you do ANY work for either pay or profit?”. Those who responded “No” were assigned as unemployed (=1) in the past week. The housing instability was obtained from two questionnaires: 1) “Is this household currently caught up on rent payments?” and 2) “Is this household currently caught up on mortgage payments?”. Those who responded “No” to either of the questions were assigned as having housing instability (=1). Food insecurity was measured using the question, “In the last 7 days, which of these statements best describes the food eaten in your household?” Respondents who reported “sometimes not enough to eat”, and “often not enough to eat” were categorized as food insecure (=1) while those who selected “enough of the kinds of food (I/we) wanted to eat”, or “enough, but not always the kinds of food (I/we) wanted to eat” were coded as food secure (=0).

Next, we note how the disability measure was captured. Disability was derived from a set of six questions on difficulty 1) seeing, 2) hearing, 3) remembering or concentrating, 4) walking or climbing stairs, 5) self-care, and 6) communicating. The following responses, “Yes – a lot of difficulty” or “Cannot do at all”, were coded as having the disability in severe form (=1), whereas the responses, “No – no difficulty” or “Yes – some difficulty” were coded as not having the disability in severe form (=0).

© 2025 Graff M and Aung TW. *JAMA Network Open*.

Lastly, we provide additional details into the policy protection from utility disconnection measure. Approximately 2% of the HPS data had partial coverage, meaning a protection was in place for part of the time during the data collection period. For example, Connecticut's winter protection period extends from November 1 through May 1, but in 2023 the HPS collected responses between April 26 and May 8. Because only a subset (April 26–30) fell within the policy period, while others (May 1–8) did not, we conservatively coded these observations as "not covered." This approach ensures that protections are not overstated in the analysis. We further conducted sensitivity analysis to check whether assigning the partial coverage as yes/no (coverage under the disconnection policy) impacted our estimates. We did not find any differences and thus, again to be conservative, assigned any state and time-period with partial coverage as not covered under the disconnection policy.

In 2022, there were no instances of partial coverage. In 2023, states that have partial coverage include Illinois (policy spans December 1 – March 31 but data collection ran from March 29-April 10, 2023); Iowa (policy spans November 1 – April 1 but data collection ran from March 29-April 10, 2023); Kentucky (policy spans November 1 – March 31 but data collection ran from March 29-April 10, 2023); Michigan (policy spans November 1 – March 31 but data collection ran from March 29-April 10, 2023); Minnesota (policy spans October 1 – April 30 but data collection ran from April 26-May 8, 2023 and September 20-October 2, 2023); Mississippi (policy spans December 1 – March 1 but data collection ran from March 1-March 13, 2023); Montana (policy spans November 1 – April 1 but data collection ran from March 29-April 10, 2023); New Hampshire (policy spans November 15 – March 31 but data collection ran from March 29-April 10, 2023); North Carolina (policy spans November 1 – March 31 but data

© 2025 Graff M and Aung TW. *JAMA Network Open*.

collection ran from March 29-April 10, 2023); Pennsylvania (policy spans December 1 – March 31 but data collection ran from March 29-April 10, 2023); South Carolina (policy spans December 1 – March 31 but data collection ran from March 29-April 10, 2023); South Dakota (policy spans November 1 – March 31 but data collection ran from March 29-April 10, 2023); and Vermont (policy spans November 1 – March 31 but data collection ran from March 29-April 10, 2023);.

In 2024, HPS data collection periods were slightly different than in 2023. Therefore, Idaho (policy spans December – February but data collection ran from February 6-March 4, 2024); Indiana (policy spans December 1 – March 15 but data collection ran from March 5-April 1, 2024); Massachusetts (policy spans November 15 – March 15 but data collection ran from March 5-April 1, 2024); New Jersey (policy spans November 15 – March 15 but data collection ran from March 5-April 1, 2024); New Mexico (policy spans November 15 – March 15 but data collection ran from March 5-April 1, 2024); New York (policy spans November 15 – April 15 but data collection ran from April 2-April 29, 2024); Ohio (policy spans November 15 – April 15 but data collection ran from April 2-April 29, 2024); Rhode Island (policy spans November 15 – March 15 but data collection ran from April 2-April 29, 2024); Washington (policy spans November 15 – March 15 but data collection ran from March 5-April 1, 2024); and Wisconsin (policy spans November 1 – April 15 but data collection ran from April 2-April 29, 2024) had partial coverage at one point during the data collection period.

Overall, cases of partial coverage occurred when the HPS collection window slightly overlapped with the beginning or end of state moratorium periods, most often in late March–early April or

© 2025 Graff M and Aung TW. *JAMA Network Open*.

late April–early May. These instances were concentrated in states with winter shutoff protections, where moratoria typically extend through March or April.

© 2025 Graff M and Aung TW. *JAMA Network Open*.

eTable 1. Multivariable Analyses of Association Between Energy Insecurity Components and Mental Health Symptoms

Characteristic[a]	Depression symptoms[b]		Anxiety symptoms[c]	
	Adjusted Odds Ratios [95% CI]	p-value[d]		p-value[d]
**Energy related social determinants of health**				
Unable to pay energy bill		0.000		0.000
No	1 [Reference]		1 [Reference]	
Yes	**1.27 [1.24,1.30]**		1.28 [1.24,1.31]	
Kept house at unsafe temperature		0.000		0.000
No	1 [Reference]		1 [Reference]	
Yes	1.60 [1.56,1.63]		1.54 [1.50,1.57]	
Forego expenses to pay energy bill		0.000		0.000
No	1 [Reference]		1 [Reference]	
Yes	1.74 [1.69,1.78]		1.79 [1.74,1.84]	
**Other social determinants of health**				
Unemployed past week		0.000		0.000
No	1 [Reference]		1 [Reference]	
Yes	1.22 [1.19,1.25]		1.15 [1.12,1.18]	
Housing unstable		0.000		0.000
No	1 [Reference]		1 [Reference]	
Yes	1.25 [1.21,1.31]		1.30 [1.25,1.35]	
Food insecure past week		0.000		0.000
No	1 [Reference]		1 [Reference]	
Yes	1.77 [1.71,1.84]		1.79 [1.72,1.86]	
**Age**		0.000		0.000
18-34 years old	1 [Reference]		1 [Reference]	
35-49 years old	0.70 [0.67,0.72]		0.71 [0.69,0.73]	
50-64 years old	0.45 [0.43,0.46]		0.43 [0.42,0.45]	
65+ years old	0.25 [0.24,0.26]		0.22 [0.22,0.23]	
**Sex**		0.000		0.000
Female	0.95 [0.93,0.98]		1.36 [1.33,1.39]	
Male	1 [Reference]		1 [Reference]	
**Race & ethnicity**[e]		0.000		0.000
Hispanic	0.76 [0.72,0.79]		0.68 [0.66,0.70]	
Non-Hispanic, Asian	0.80 [0.76,0.83]		0.55 [0.53,0.57]	
Non-Hispanic, Black	0.69 [0.66,0.73]		0.57 [0.55,0.60]	
Non-Hispanic, White	1 [Reference]		1 [Reference]	
Non-Hispanic, Other[f]	0.99 [0.94,1.04]		0.92 [0.88,0.96]	
**Education**		0.000		0.000
High school or less	1 [Reference]		1 [Reference]	
Some college or associate's degree	1.05 [1.02,1.09]		1.18 [1.15,1.21]	
Bachelor's/Graduate degree	0.84 [0.81,0.86]		1.07 [1.05,1.10]	
**Annual household income ($)**		0.000		0.000
<35 000	1 [Reference]		1 [Reference]	
35 000 - 74 999	0.96 [0.93,0.99]		0.98 [0.95,1.01]	
75 000 - 149 999	0.89 [0.86,0.92]		0.94 [0.91,0.97]	
>150 000	0.64 [0.61,0.67]		0.76 [0.72,0.79]	

© 2025 Graff M and Aung TW. *JAMA Network Open*.

**Marital status**		0.000		0.000
Now married	1 [Reference]		1 [Reference]	
Widowed/Divorced/Separated	1.32 [1.28,1.35]		1.18 [1.14,1.21]	
Never married	1.39 [1.35,1.43]		1.23 [1.19,1.26]	
**Household size**		0.000		0.000
1-2 persons	1 [Reference]		1 [Reference]	
3-4 persons	1.06 [1.04,1.09]		1.06 [1.03,1.09]	
5 or more persons	1.16 [1.10,1.22]		1.10 [1.05,1.15]	
**Children in home**		0.000		0.000
No child in home	1 [Reference]		1 [Reference]	
1-2 children	0.80 [0.77,0.82]		0.87 [0.84,0.89]	
3 or more children	0.65 [0.61,0.70]		0.76 [0.72,0.80]	
**House type**		0.000		0.000
Detached one-family	1 [Reference]		1 [Reference]	
Attached one-family	0.96 [0.92,1.01]		0.99 [0.96,1.03]	
Apartment	1.07 [1.03,1.11]		1.10 [1.06,1.14]	
Other (mobile home, boat, RV, van)	0.99 [0.95,1.03]		0.98 [0.94,1.01]	
**Home ownership**		0.02		0.001
Owner	1 [Reference]		1 [Reference]	
Renter	1.05 [1.01,1.09]		1.07 [1.03,1.11]	
**Disability**		0.000		0.000
No disability	1 [Reference]		1 [Reference]	
1 severe disability	3.00 [2.92,3.08]		2.82 [2.75,2.90]	
2 or more severe disability	6.58 [6.23,6.94]		5.89 [5.54,6.26]	
**Census region**		0.000		0.000
Northeast	1 [Reference]		1 [Reference]	
South	1.10 [1.06,1.14]		1.06 [1.03,1.10]	
Midwest	1.00 [0.96,1.04]		0.97 [0.94,1.00]	
West	1.05 [1.01,1.09]		1.04 [1.01,1.07]	
**Policy protection from utility disconnection**		0.45		0.49
No	1 [Reference]		1 [Reference]	
Yes	0.98 [0.94,1.03]		0.99 [0.95,1.02]	
Unweighted number of observations	1,139,607		1,139,607	
Weighted number of observations	187,356,336		187,356,336	

[a] All variables listed are included in the multivariable analyses.

[b] Depression symptoms over the last two weeks were assessed from Patient Health Questionnaire (PHQ-2): “How often have you been bothered by: 1) having little interest or pleasure in doing things; and 2) feeling down, depressed, or hopeless?”. A summed response with a total score of three or greater was considered a positive screen for major depressive disorder.

[c] Anxiety symptoms over the last two weeks were assessed from Generalized Anxiety Disorder (GAD-2): “How often have you been bothered by: 1) feeling nervous, anxious, or on edge; and 2) not being able to stop or control worrying?”. A summed response with a total score of three or greater was considered a positive screen for generalized anxiety disorder.

[d] P-value from adjusted Wald test.

[e] Self-reported by participants

[f] Includes American Indian or Alaska Native, Native Hawaiian, Chamorro, Samoan, Other Pacific Islander, or multiple races.

© 2025 Graff M and Aung TW. *JAMA Network Open*.

eTable 2. Multivariable Analyses of Association Between Composite Energy Insecurity Indicator and Mental Health Symptoms

Characteristic[a]	Depression symptoms[b]		Anxiety symptoms[c]	
	Adjusted Odds Ratios [95% CI]	p-value[d]		p-value[d]
**Energy related social determinants of health**				
Composite energy insecure indicator[e]		0.000		0.000
No	1 [Reference]		1 [Reference]	
Yes	2.31 [2.26,2.37]		2.29 [2.24,2.34]	
**Other social determinants of health**				
Unemployed past week		0.000		0.000
No	1 [Reference]		1 [Reference]	
Yes	1.22 [1.18,1.25]		1.14 [1.12,1.17]	
Housing unstable		0.000		0.000
No	1 [Reference]		1 [Reference]	
Yes	1.34 [1.29,1.40]		1.39 [1.34,1.44]	
Food insecure past week		0.000		0.000
No	1 [Reference]		1 [Reference]	
Yes	2.05 [1.97,2.13]		2.07 [1.99,2.15]	
**Age**		0.000		0.000
18-34 years old	1 [Reference]		1 [Reference]	
35-49 years old	0.71 [0.69,0.73]		0.72 [0.70,0.74]	
50-64 years old	0.46 [0.44,0.47]		0.44 [0.43,0.46]	
65+ years old	0.26 [0.25,0.27]		0.22 [0.22,0.23]	
**Sex**		0.000		0.000
Female	0.94 [0.92,0.96]		1.34 [1.31,1.37]	
Male	1 [Reference]		1 [Reference]	
**Race & ethnicity**[f]		0.000		0.000
Hispanic	0.76 [0.73,0.79]		0.68 [0.66,0.71]	
Non-Hispanic, Asian	0.80 [0.76,0.84]		0.55 [0.53,0.58]	
Non-Hispanic, Black	0.69 [0.65,0.72]		0.57 [0.55,0.59]	
Non-Hispanic, White	1 [Reference]		1 [Reference]	
Non-Hispanic, Other[g]	1.00 [0.96,1.05]		0.93 [0.89,0.98]	
**Education**		0.000		0.000
High school or less	1 [Reference]		1 [Reference]	
Some college or associate's degree	1.07 [1.03,1.10]		1.19 [1.16,1.22]	
Bachelor's/Graduate degree	0.85 [0.83,0.88]		1.08 [1.06,1.11]	
**Annual household income ($)**		0.000		0.000
<35 000	1 [Reference]		1 [Reference]	
35 000 - 74 999	0.95 [0.92,0.98]		0.97 [0.94,1.00]	
75 000 - 149 999	0.87 [0.84,0.90]		0.92 [0.89,0.95]	
>150 000	0.63 [0.59,0.66]		0.74 [0.71,0.77]	
**Marital status**		0.000		0.000
Now married	1 [Reference]		1 [Reference]	
Widowed/Divorced/Separated	1.32 [1.29,1.36]		1.19 [1.15,1.22]	
Never married	1.38 [1.34,1.42]		1.22 [1.18,1.25]	
**Household size**		0.000		0.000
1-2 persons	1 [Reference]		1 [Reference]	
3-4 persons	1.07 [1.04,1.10]		1.06 [1.03,1.10]	
5 or more persons	1.18 [1.12,1.24]		1.11 [1.06,1.16]	

© 2025 Graff M and Aung TW. *JAMA Network Open*.

**Children in home**		0.000		0.000
No child in home	1 [Reference]		1 [Reference]	
1-2 children	0.78 [0.76,0.81]		0.85 [0.83,0.88]	
3 or more children	0.64 [0.60,0.68]		0.74 [0.70,0.79]	
**House type**		0.000		0.000
Detached one-family	1 [Reference]		1 [Reference]	
Attached one-family	0.96 [0.91,1.00]		0.99 [0.95,1.02]	
Apartment	1.06 [1.02,1.09]		1.09 [1.05,1.12]	
Other (mobile home, boat, RV, van)	1.00 [0.96,1.05]		0.99 [0.96,1.03]	
**Home ownership**		0.05		0.003
Owner	1 [Reference]		1 [Reference]	
Renter	1.04 [1.00,1.08]		1.06 [1.02,1.10]	
**Disability**		0.000		0.000
No disability	1 [Reference]		1 [Reference]	
1 severe disability	3.04 [2.95,3.12]		2.86 [2.79,2.93]	
2 or more severe disability	6.81 [6.45,7.18]		6.09 [5.74,6.46]	
**Census region**		0.000		0.000
Northeast	1 [Reference]		1 [Reference]	
South	1.09 [1.05,1.13]		1.06 [1.02,1.09]	
Midwest	0.99 [0.95,1.03]		0.96 [0.94,0.99]	
West	1.04 [1.01,1.08]		1.03 [1.00,1.06]	
**Policy protection from utility disconnection**		0.34		0.37
No	1 [Reference]		1 [Reference]	
Yes	0.98 [0.94,1.02]		0.98 [0.95,1.02]	
Unweighted number of observations	1,139,607		1,139,607	
Weighted number of observations	187,356,336		187,356,336	

[a] All variables listed are included in the multivariable analyses.

[b] Depression symptoms over the last two weeks were assessed from Patient Health Questionnaire (PHQ-2): "How often have you been bothered by: 1) having little interest or pleasure in doing things; and 2) feeling down, depressed, or hopeless?". A summed response with a total score of three or greater was considered a positive screen for major depressive disorder.

[c] Anxiety symptoms over the last two weeks were assessed from Generalized Anxiety Disorder (GAD-2): "How often have you been bothered by: 1) feeling nervous, anxious, or on edge; and 2) not being able to stop or control worrying?". A summed response with a total score of three or greater was considered a positive screen for generalized anxiety disorder.

[d] P-value from adjusted Wald test.

[e] Composite energy insecure indicator is defined as having any of the three energy insecure components (unable to pay energy bill; kept house at unsafe temperature; and forgo expenses to pay energy bills).

[f] Self-reported by participants.

[g] Includes American Indian or Alaska Native, Native Hawaiian, Chamorro, Samoan, Other Pacific Islander, or multiple races.

© 2025 Graff M and Aung TW. *JAMA Network Open*.
