# Supplementary material for: Energy Insecurity and Mental Health Symptoms in US Adults
Source: JAMA Netw Open. 2025 Oct 27;8(10):e2539479. doi: 10.1001/jamanetworkopen.2025.39479 (PMC12559964; doi:10.1001/jamanetworkopen.2025.39479)
Supplement: Supplement 2. — Data Sharing Statement [file jamanetwopen-e2539479-s002.pdf]

Data Sharing Statement

Graff. Energy Insecurity and Mental Health Symptoms in US Adults. *JAMA Netw Open*.
Published October 27, 2025. doi:10.1001/jamanetworkopen.2025.39479

Data
Data available: Yes
Data types: Deidentified participant data
How to access data: De-identified Household Pulse Survey data are publicly available at https://www.census.gov/programs-surveys/household-pulse-survey/data/datasets.html.
When available: With publication

Supporting Documents
Document types: None

Additional Information
Who can access the data: Anyone requesting the data.
Types of analyses: For any purpose.
Mechanisms of data availability: With investigator support.
